# Oxidative stress impairs the meat quality of broiler by damaging mitochondrial function, affecting calcium metabolism and leading to ferroptosis

**DOI:** 10.5713/ab.22.0019

**Published:** 2022-05-02

**Authors:** Zuodong Chen, Tong Xing, Jiaolong Li, Lin Zhang, Yun Jiang, Feng Gao

**Affiliations:** 1 College of Animal Science and Technology, Jiangsu Key Laboratory of Animal Origin Food Production and Safety Guarantee, Jiangsu Collaborative Innovation Center of Meat Production and Processing, Quality and Safety Control, Joint International Research Laboratory of Animal Health and Food Safety, National Experimental Teaching Demonstration Center of Animal Science, Nanjing Agricultural University, Nanjing 210095, China; 2 School of Food Science and Pharmaceutical Engineering, Nanjing Normal University, Nanjing 210023, China

**Keywords:** Broiler, Calcium Metabolism, Ferroptosis, Meat Quality, Mitochondria, Oxidative Stress

## Abstract

**Objective:**

This work was conducted to investigate the effects of oxidative stress on meat quality, mitochondrial function, calcium metabolism and ferroptosis of broilers.

**Methods:**

In this study, a total of 144 one-day-old male Ross 308 chicks were divided into 3 groups (control group, saline group, and hydrogen peroxide [H_2_O_2_] group) with 6 replicates of 8 broilers each. The study lasted for 42 d. The broilers in the saline and H_2_O_2_ groups were intraperitoneally injected with 0.75% saline and 10.0% H_2_O_2_ on the 16th and 37th day of the experimental period respectively, the injection volumes were 1.0 mL/kg of broiler body weight. On the 42nd day of the experimental period, two chicks were randomly selected from each cage, a total of thirty-six chicks were stunned by electric shock and slaughtered to collect breast muscle samples.

**Results:**

The H_2_O_2_ exposure reduced pH value, increased drip loss and shear force of breast meat (p<0.05), impaired the ultrastructure and function of mitochondria. The H_2_O_2_ exposure damaged the antioxidant system in mitochondria, excessive reactive oxygen species carbonylation modified calcium channels on mitochondria, which impaired the activities of key enzymes on calcium channel, resulted in the increased calcium concentration in cytoplasm and mitochondria (p<0.05). In addition, the H_2_O_2_ exposure increased the iron content and lipid peroxidation (p<0.05), which induced ferroptosis.

**Conclusion:**

Oxidative stress could impair meat quality by causing mitochondrial dysfunction, resulting in calcium metabolism disorder and ferroptosis.

## INTRODUCTION

Popular price, high nutritional values, cultural acceptance, and even religious beliefs are the reasons for the growing global broiler meat consumption [1]. Nowadays, intensive farming genetically selected broilers and even global warming lead to oxidative stress, which has become a crucial environment problem threatening the poultry meat industry [2]. Oxidative stress leads to the decline of production performance and meat quality of broilers, causing huge economic losses to producers, and the poor quality meat also brings health risks to consumers [3,4]. Oxidative stress is an important mechanism of biological damage, which affects the growth performance and meat quality of broilers [5,6]. Previous study had reported that oxidative stress could increase shear force and decrease pH value in the breast meat of broilers [5]. However, the effects of oxidative stress on broiler meat quality and its molecular mechanisms are still unclear.

The main cause of oxidative stress is the imbalance between excessive reactive oxygen species (ROS) and the antioxidant capacity of organism. ROS are mainly produced in mitochondrial respiratory chain and their excessive production reverse attacks the mitochondrial respiratory chain, resulting in mitochondrial dysfunction [7]. Intraperitoneal injection of hydrogen peroxide (H_2_O_2_) may cause oxidative stress by directly increasing the levels of ROS *in vivo*, resulting in mitochondrial damage [8]. Mitochondrial function plays an essential role in calcium metabolism, which includes calcium transport. As an important second messenger, calcium participates in the internal and external signal transmission of muscle cells, which leads to the muscle contraction. But the oxidative stress could cause the damage to calcium homeostasis in broiler muscle, furthermore harm to quality of broiler meat during transformation process from postmortem muscle to edible meat [9]. Nowadays, the influence of calcium metabolism on meat quality has been widely accepted.

Ferroptosis is a newly described mode of cell death, which is closely related to mitochondrial dysfunction and different from other cell death modes in morphology and biochemistry [10]. In morphology, the cell membrane is intact and shrunk, the volume of mitochondria is reduced, the cristae of mitochondria are reduced or disappeared, and the outer membrane of mitochondria is broken [10]. In a biochemical aspect, glutathione (GSH) is exhausted, the activity of glutathione peroxidase 4 (GPX4) is decreased, therefore the lipid oxides are not metabolized by glutathione reduction reaction catalyzed by GPX4, and then the divalent iron ions oxidize lipid by Fenton reaction to produce a large number of ROS, which promotes ferroptosis of cells [11].

In the present study, we aimed for studying the relationship among meat quality, calcium metabolism and ferroptosis in the broiler breast muscle, which could provide scientific evidence for explaining the mechanism of oxidative damage.

## MATERIALS AND METHODS

### Broiler management

All experimental considerations procedures and experimental animals used in the experiment were consulted by the experimental animal management committee of Nanjing Agricultural University, Nanjing, P.R. China (GB/T 35892-2018). A total of 144 one-day-old male Ross 308 chicks, with a similar initial body weight, were randomly divided into 3 treatments, 6 cages (replicates) per treatment, 8 chicks per cage (120 cm×65 cm×55 cm). The 3 experimental groups included: i) control group, the birds were free to get basal diet and water without injection, ii) saline group, birds were free to get basal diet and water, and saline buffer (0.75%) was injected into the abdominal cavity of the birds, and iii) H_2_O_2_ group, birds were free to get basal diet and water, and H_2_O_2_ buffer (10.0%) was injected into the abdominal cavity of the birds. The injection dosages of saline and H_2_O_2_ were 1.0 mL/kg broiler body weight, and the H_2_O_2_ were dissolved in physiological saline. The injections were carried out on the 16th and 37th days of the experimental period [8]. The basal diet (Table 1) met the nutrient recommendations of Ross 308 broilers. The photoperiod and environmental temperature met the procedures of broiler production and broiler welfare.

### Sample collection

On the 42nd day of the experimental period, two chickens per cage (close to the average weight), a total of thirty-six chicks were stunned by electric shock (50 V, alternating current, 400 Hz, 5 s), bloodletting was performed immediately after stunned by electric shock, and the pectoral muscles were collected by autopsy. The equipment used to electric shock the chickens was an electric anesthesia machine (SQ05 poultry stunner; Wujiang Aneng Electronic Technology Co., Ltd, Suzhou, China). The entire left pectoral muscle was placed into a vacuum bag and stored in the 4°C refrigerator to determine the meat quality. On the right side of pectoral muscle, muscle slices were immediately frozen in liquid nitrogen for subsequent experimental analysis. Fresh pectoral muscle was collected and immediately extracted the mitochondria using the mitochondrial extraction kit (Beyotime Biotechnology Co., Ltd, Shanghai, China). For ultrastructural observation, a piece of right pectoral muscle was sliced into 1.5 mm^3^ and fixed in 2.5% glutaraldehyde solution.

### Measurement of meat quality

At 24 h post mortem of broiler, the pH, lightness (L*), redness (a*), and yellowness (b*) and drip loss of meat were measured according to the method of Pan et al [12]. The determination of meat cooking loss was measured by the method of Fellenberg and Speisky [13]. For the shear force determination, the method according to Lin et al [3].

### Ultrastructural observation

The experimental method was referred to Pan et al [12]. The fixed samples were washed with phosphate buffer, then put into 1% osmium tetroxide solution (v/v). After dehydration, the samples were embedded in Epon 812. The cut slices were placed on the copper mesh, stained with uranium acetate and lead citrate for observation. Mitochondrial morphological changes were observed by transmission electron microscopy (HT7700; Hitachi, Tokyo, Japan).

### Determination of mitochondrial reactive oxygen species

Mitochondria were isolated from fresh breast muscle samples by a commercial kit (C3601; Beyotime, Shanghai, China), according to manufacturer instructions. The 2′7′-dichlorodihydrofluorescein diacetate (DCFH-DA) was oxidized by ROS to form a green, fluorescent substance. Under a fluorescence microscope, the intracellular fluorescence intensity was proportional to the levels of ROS.

### Mitochondrial membrane potential measurement

Mitochondrial membrane potential (MMP) measurement kit with JC-1 (G009; Jiancheng Bioengineering Institute, Nanjing, China) was used to detect the MMP changes in each mitochondrion of the breast muscle tissues, according to the manufacturer’s instructions.

### Measurement of mitochondrial complexes and redox parameters

Muscle mitochondria were homogenized in chilled phosphate saline buffer and then centrifuged at the speed of 1,500 rpm for 10 min at 4°C. The supernatant was obtained for the evaluation of mitochondrial oxidation parameters. The activities of mitochondrial complex I and complex III, the contents of protein carbonyl and malondialdehyde (MDA), the activities of total antioxidant capacity (T-AOC), total superoxide dismutase (T-SOD), and glutathione peroxidase (GSH-Px) were detected by commercial kits (Jiancheng Bioengineering Institute, China), all the operations according to instructions.

### Determination of glutathione and oxidized glutathione

The activities of GSH and oxidized glutathione (GSSG) were detected by a commercial kit (Jiancheng Bioengineering Institute, China), following the manufacturer’s instructions. GSH = total GSH–2×GSSG.

### Determination of Ca^2+^ and Fe^3+^ concentration

The concentrations of Ca^2+^ and Fe^3+^ were assayed by the modified method of Parrish Jr et al [14]. 2.5 g breast muscle was homogenized (985370EUR; BioSpec, San Diego, CA, USA) with 12.5 mL cold solution (2 mM adenosine 5′-triphosphate and MgCl) at 11,000 rpm for 1 min. Removed supernatants, added 2 mL 5% trichloroacetic acid and 0.5% strontium chloride mixed solution, centrifuged at 1,600 g for 10 min. Finally, extracted supernatants were determined by atomic absorption spectrometry (170-10; Hitachi Co., Japan).

### Terminal deoxynucleotidyl transferase mediated dUTP nick end labeling analysis

The terminal deoxynucleotidyl transferase mediated dUTP nick end labeling (TUNEL) analysis was performed according to the instructions of the manufacturer of the Bright Green Apoptosis Detection Kit A112 (Vazyme, Nanjing, China). Briefly, the muscle paraffin sections were dewaxed by the following steps: 100% xylene for 15 min, 100% ethanol for 5 min, 95% ethanol for 5 min, 85% and 75% ethanol for 5 min respectively. The muscle sections were incubated with proteinase K at 37°C for 10 min, and then incubated with a labeling mixture consisting of 2′-deoxyuridine 5′-triphosphate (dUTP) in the terminal deoxynucleotidyl transferase (TdT) at 37°C for 2 h. Subsequently, the muscle sections were incubated with 4′,6-diamidino-2-phenylindole (DAPI) for 15 min without light. Finally, the sections were rinsed with phosphate-buffered saline and mounted by an antifade mounting medium. The evaluation of muscle tissues apoptosis was achieved via a fluorescence microscope.

### RNA extraction and gene expression analysis

Total RNA from the breast muscle samples were isolated using TRIzol reagent (Takara Biotechnology Co. Ltd, Dalian, China). The library of cDNA was performed using Prime Script RT Master Mix (Takara Biotechnology Co. Ltd, China). The real-time polymerase chain reaction was performed using an ABI PRISM 7500 Detection System (Applied Biosystems, Foster City, CA, USA). The primer sequences were shown in Table 2, and the gene expression was calculated using the method of Chen et al [8].

### Western blot analysis

Frozen samples of breast muscle were homogenized to collect the supernatants. The supernatants were electrophoresed, transferred to the membrane, incubated, and photographed. The antibodies of sarcoplasmic/endoplasmic reticulum calcium ATPase 1 (SERCA1 #12293), mitochondrial calcium uniporter (MCU #14997), transferrin receptor 1 (TFR1 #13113), GPX4 (#52455), solute carrier family 7 member 11 (SLC7A11 #98051) and horseradish-peroxidase-conjugated (#7074) were produced by Cell Signaling Technology (Beverly, MA, USA). The antibodies of plasma membrane Ca^2+^-ATPase (PMCA bs-4978R), transient receptor potential channel 1 (TRPC1 bs-10404R), and sodium-calcium exchanger (NCX bs-1550R) were produced by Bioss biotechnology company (Beijing, China). The antibody of glyceraldehyde-3-phosphate dehydrogenase (GAPDH GB11002) was produced by the Servicebio biotechnology company (Wuhan, Hubei, China). Incubation of the membrane with electrochemiluminescence reagents (ECL; EpiZyme Inc., Shanghai, China). Quantitative analysis of the optical density of protein bands by Imager Bio-Rad ChemiDoc Touch and Image Quantity One software (GE, Uppsala, Sweden). According to the density of the reference bands, the band density between different spots was normalized, according to the method of Dixon et al [10].

### Measurement of carbonyl protein content

Detection of carbonylation degree of MCU and SERCA1, the immunoprecipitation (IP) method was used to precipitate the protein. Experimental operation steps: appropriate amount of protein beads were taken out (beads:protein = 1 μL:14 μg), washed with radio-immunoprecipitation assay buffer (RIPA). Combined beads with antibodies and reacted at 4°C for at least 4 hours. 50 μL of protein sample (1 μg/μL) were taken out, added the appropriate amount of beads and RIPA buffer to make the total reaction volume 200 μL, and reacted overnight at 4°C. After the reaction was over, washed the beads with RIPA buffer. The volume of protein sample was 20 μL, the concentration of protein sample was 1 μg/μL, and the protein sample was taken into centrifuge tube. Protein carbonylation detection used the protein oxidation detection kit (Merck Biotechnology Company Ltd., Kenilworth, NJ, USA), all the operations according to instructions.

### Statistical analyses

Data were analyzed with the use of SPSS 20.0 (SPSS, Inc., Chicago, IL, USA). One-way analysis of variance analyses were used to assess differences. The data were described as means±standard error, and differences were considered significant at p<0.05.

## RESULTS

### Meat quality

As shown in Table 3, after exposure to H_2_O_2_, the value of pH_24 h_ was decreased in the H_2_O_2_ treatment than that in the control and the saline treatments (p<0.05). The contents of drip loss and shear force were higher in the H_2_O_2_ treatment than those in the other two treated groups (p<0.05). Among the three treatments, there were no differences of a*, b*, L* and cooking loss in broiler breast meat (p>0.05).

### Mitochondrial structure

**Figure 1:**
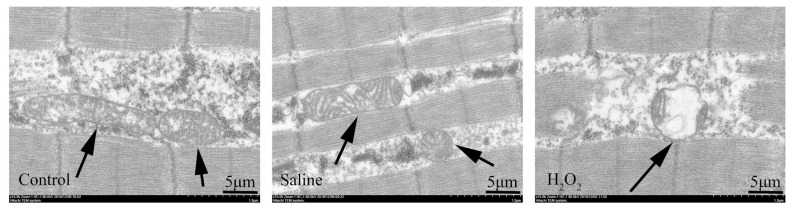
Effects of H_2_O_2_ exposure on the ultrastructural morphology in the breast muscles. Scale bar: 1 μm. The control group was the non-injected treatment. The saline group: birds were injected with physiological saline buffer (0.75%) with a dosage of 1.0 mL/kg BW. The H_2_O_2_ group: birds were given an injection of 10.0% H_2_O_2_ with a dosage of 1.0 mL/kg BW. BW, body weight.

As shown in Figure 1, the observation of mitochondrial ultrastructure images indicated that H_2_O_2_ exposure significantly changed the morphological structure of mitochondria. After H_2_O_2_ exposure, the mitochondria in the H_2_O_2_ group were crenated, the cristae were dissolved, and the matrix electron density was decreased. In the control and saline groups, the structure of mitochondria was complete, and the cristae of mitochondria was abundant.

### Mitochondrial function and redox state

**Figure 2:**
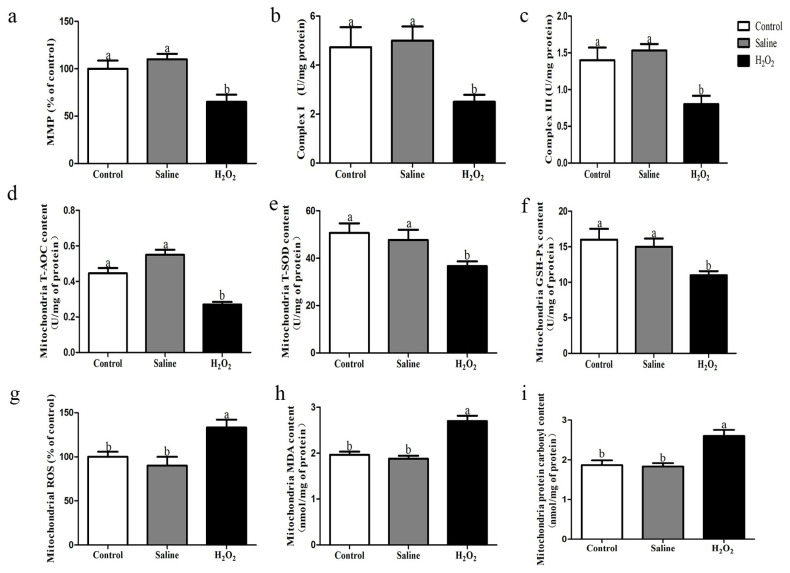
(a–c) Effects of H_2_O_2_ exposure on the level of mitochondrial membrane potential, and activities of complex I and complex III in muscle mitochondria. (d–i) Effects of H_2_O_2_ exposure on the activities of T-AOC, T-SOD, GSH-Px, and levels of ROS, MDA, and protein carbonyl in muscle mitochondria. The control group was the non-injected treatment. The saline group: birds were injected with physiological saline buffer (0.75%) with a dosage of 1.0 mL/kg BW. The H_2_O_2_ group: birds were given an injection of 10.0% H_2_O_2_ with a dosage of 1.0 mL/kg BW. Data were reported as mean±standard error (n = 6), T-AOC, total antioxidant capacity; T-SOD, total superoxide dismutase; GSH-Px, glutathione peroxidase; ROS, reactive oxygen species; MDA, malondialdehyde; BW, body weight. ^a,b^ denoted p<0.05.

As shown in Figure 2. The levels of mitochondrial ROS in the H_2_O_2_ group were higher than those in the control and saline groups (p<0.05, Figure 2g). In addition, treatment with H_2_O_2_ reduced the level of MMP, and the activities of complex I and complex III (p<0.05; Figure 2a–c). After H_2_O_2_ exposure, the mitochondrial activities of T-AOC, T-SOD, and GSH-Px in the H_2_O_2_ group were lower when compared to those in the control and saline groups (p<0.05; Figure 2d, f). Additionally, the contents of MDA and protein carbonyl in the H_2_O_2_ group were higher than those of the other two groups (p<0.05, Figure 2h, i).

### Concentration of Ca^2+^ and relative protein expression levels of Ca^2+^ channel

**Figure 3:**
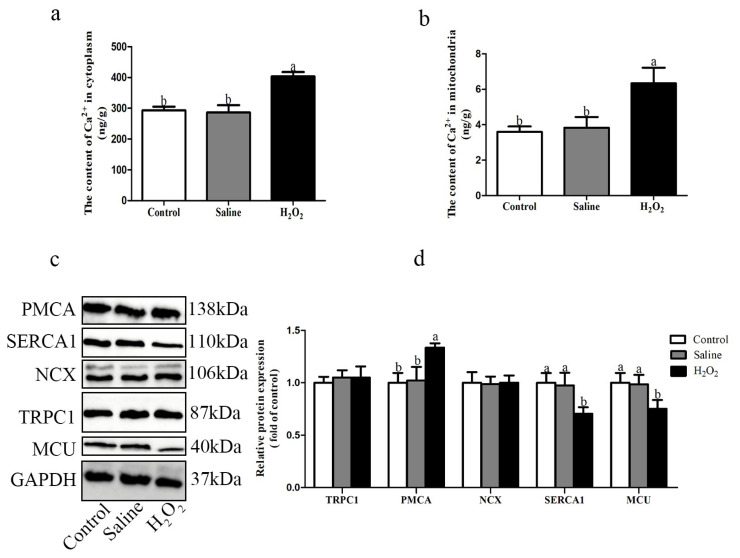
(a, b) Effects of H_2_O_2_ exposure on the contents of Ca^2+^. (c, d) Effects of H_2_O_2_ exposure on the relative protein expression levels of TRPC1, PMCA, NCX, SERCA1, and MCU. The control group was the non-injected treatment. The saline group: birds were injected with physiological saline buffer (0.75%) with a dosage of 1.0 mL/kg BW. The H_2_O_2_ group: birds were given an injection of 10.0% H_2_O_2_ with a dosage of 1.0 mL/kg BW. Data were reported as mean±standard error (n = 6). TRPC1, transient receptor potential channel 1; PMCA, plasma membrane Ca^2+^-ATPase; NCX, sodium-calcium exchanger; SERCA1, sarcoplasmic/endoplasmic reticulum calcium ATPase 1; MCU, mitochondrial calcium uniporter; BW, body weight. ^a,b^ denoted p<0.05.

According to Figure 3, compared with the control and saline groups, the contents of Ca^2+^ in cytoplasm and mitochondria were higher in the H_2_O_2_ treated group (p<0.05; Figure 3a, b). H_2_O_2_ exposure decreased the protein expression levels of SERCA1 and MCU (p<0.05; Figure 3c, d), while the protein expression level of PMCA was increased in the H_2_O_2_ treated group (p<0.05; Figure 3c, d).

### Carbonylation modification levels of SERCA1 and MCU

**Figure 4:**
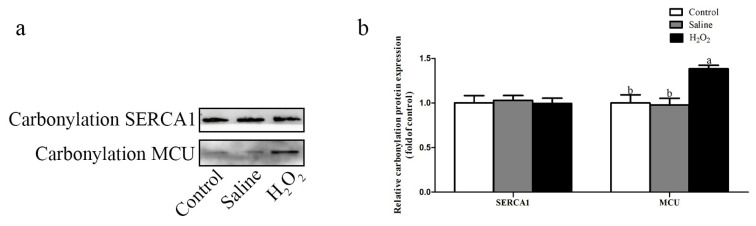
Effects of H_2_O_2_ exposure on the relative carbonylation protein expression levels of SERCA1 and MCU. The control group was the non-injected treatment. The saline group: birds were injected with physiological saline buffer (0.75%) with a dosage of 1.0 mL/kg BW. The H_2_O_2_ group: birds were given an injection of 10.0% H_2_O_2_ with a dosage of 1.0 mL/kg BW. Data were reported as mean±standard error (n = 6). SERCA1, sarcoplasmic/endoplasmic reticulum calcium ATPase 1; MCU, mitochondrial calcium uniporter; BW, body weight. ^a,b^ denoted p<0.05.

As exhibited in Figure 4, the protein carbonylation level of MCU in the H_2_O_2_ group was higher than that in the control and saline groups (p<0.05; Figure 4). No significant difference in the protein carbonylation level of SERCA1 was observed among the three groups after H_2_O_2_ exposure.

### Terminal deoxynucleotidyl transferase mediated dUTP nick end labeling analysis

**Figure 5:**
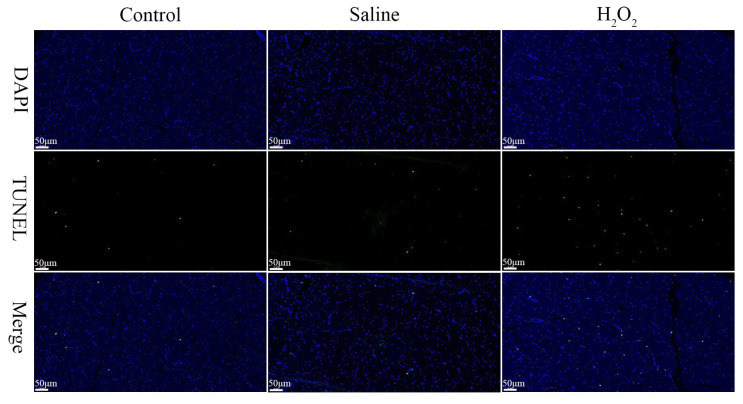
Effects of H_2_O_2_ exposure on TUNEL staining of muscle sections (Scale bar: 50 μm). The control group was the non-injected treatment. The saline group: birds were injected with physiological saline buffer (0.75%) with a dosage of 1.0 mL/kg BW. The H_2_O_2_ group: birds were given an injection of 10.0% H_2_O_2_ with a dosage of 1.0 mL/kg BW. DAPI, 4′,6-diamidino-2-phenylindole; TUNEL, TdT-mediated dUTP Nick-End Labeling; BW, body weight.

As exhibited in Figure 5, in the control, saline and H_2_O_2_ groups, the nuclei of muscle cells were stained blue, the nuclei of TUNEL-positive cells were stained green. In comparison to the control and saline groups, more TUNEL-positive cells were observed in the H_2_O_2_ group after oxidative exposure.

### Concentration of iron, GSH and GSSG

**Figure 6:**
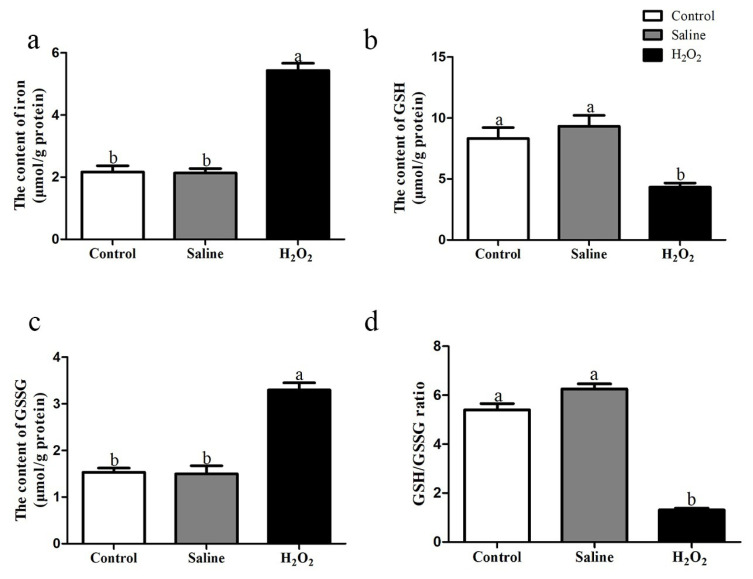
(a) Effects of H_2_O_2_ exposure on the content of iron in breast muscle. (b–c) Effects of H_2_O_2_ exposure on the contents of GSH and GSSG. (d) Effect of H_2_O_2_ exposure on the ratio of GSH/GSSG. The control group was the non-injected treatment. The saline group: birds were injected with physiological saline buffer (0.75%) with a dosage of 1.0 mL/kg BW. The H_2_O_2_ group: birds were given an injection of 10.0% H_2_O_2_ with a dosage of 1.0 mL/kg BW. Data were reported as mean±standard error (n = 6), GSH, glutathione; GSSG, oxidized glutathione; BW, body weight. ^a,b^ denoted p<0.05.

As shown in Figure 6, after oxidative exposure, the content of iron in H_2_O_2_ group was higher than that in the control and saline groups (p<0.05; Figure 6a). H_2_O_2_ treatment reduced the content of GSH and the ratio of GSH/GSSG and increased the content of GSSG (p<0.05; Figure 6b–d).

### Relative mRNA and protein expression levels of ferroptosis

**Figure 7:**
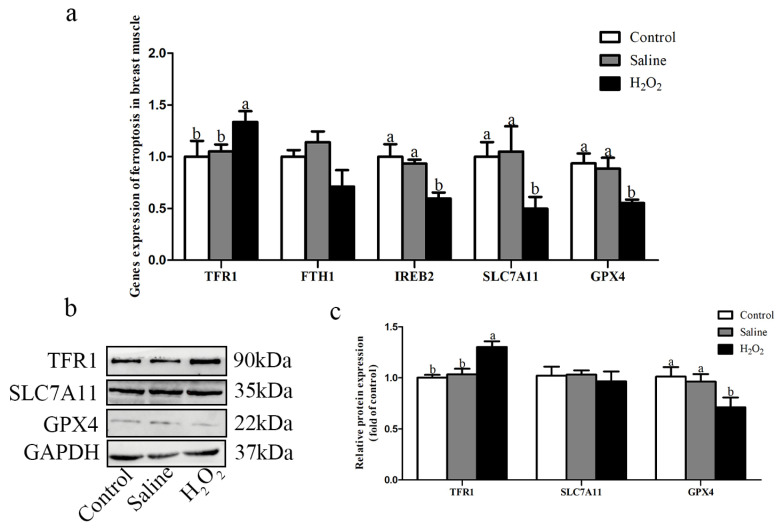
(a) Effects of H_2_O_2_ exposure on the relative mRNA expression levels of *TFR1*, *FTH1*, *IREB2*, *SLC7A11* and *GPX4*. (b, c) Effects of H_2_O_2_ exposure on the relative protein expression levels of TFR1, SLC7A11 and GPX4. The control group was the non-injected treatment. The saline group: birds were injected with physiological saline buffer (0.75%) with a dosage of 1.0 mL/kg BW. The H_2_O_2_ group: birds were given an injection of 10.0% H_2_O_2_ with a dosage of 1.0 mL/kg BW. Data were reported as mean±standard error (n = 6). *TFR1*, transferrin receptor 1; *FTH1*, ferritin heavy chain 1; *IREB2*, iron responsive element binding 2; *SLC7A11*, solute carrier family 7 member 11; *GPX4*, glutathione peroxidase 4; BW, body weight. ^a, b^ denoted p<0.05.

As shown in Figure 7, after H_2_O_2_ exposure, the mRNA expression levels of ferroptosis genes iron responsive element binding 2 (*IREB2*), *SLC7A11*, *GPX4* in the H_2_O_2_ group were down-regulated compared with those in the control and saline groups (p<0.05; Figure 7a). The mRNA expression level of *TFR1* in the H_2_O_2_ group was up-regulated than that in the other two groups (p<0.05; Figure 7a). Meanwhile, the protein expression level of TFR1 in the H_2_O_2_ group was higher than that in the other two groups, and the protein expression level of GPX4 in the H_2_O_2_ group was lower than that in the other two groups (p<0.05; Figure 7b, c).

## DISCUSSION

In the broiler industry, environmental heat stress and oxidative diet could induce the production of ROS, which attack the antioxidant defense system and cause oxidative stress. Oxidative stress has become ubiquitous, and it causes huge concerns since it reduces productivity and further impairs poultry meat quality [13]. Lu et al [15]. found that oxidative stress induced by high temperature decreased the muscle pH and increased the lightness and drip loss of breast muscle in broilers. As the main place of oxygen metabolism, mitochondria produce ROS, which lead to oxidative stress in most cells. Mujahid et al [16]. showed that oxidative stress induced by heat stress increased the content of O^2·−^ in the mitochondria of chicken skeletal muscle. The impairment of mitochondrial function would lead to the disorder of calcium metabolism and the activation of endogenous apoptosis, which was an important factor in the decline of meat quality [17]. In the present study, H_2_O_2_ exposure increased the drip loss and shear force, and decreased the value of pH_24 h_ in broiler breast meat. These above results indicated that oxidative stress deteriorated the meat quality of broilers.

When oxidative stress occurs, excessive ROS first attack the mitochondrial respiratory chain, since their membranes consist of phospholipids and proteins [18]. The principal sites of endogenous ROS production in mitochondria are complex I and III. Previous studies have reported that oxidative damage of the mitochondrial respiratory chain by exogenous ROS attack may further increase the production of endogenous ROS and lead to a vicious cycle of continuous increase of ROS [19]. Therefore, excessive ROS may in turn attack mitochondria and destroy the integrity of the mitochondrial membrane, and eventually lead to mitochondrial dysfunction. In particular, the destruction of the mitochondrial structure observed may be due to the oxidative damage of phospholipids, which accelerated the oxidation process and promoted the generation of ROS [20]. Lu et al [15] found that ROS caused mitochondrial dysfunction, affected the antioxidant function and aerobic respiration of pectoral muscle cells, and eventually reduced the pH and increased the drip loss of breast meat. In the present research, the results of the ultrastructure changes indicated that mitochondrial cristae decreased or disappeared, and mitochondrial vacuoles were severely damaged. Meanwhile, we also observed that the activities of complex I and III were decreased, and the level of MMP was down-regulated after H_2_O_2_-treatment. MMP, mitochondrial complex I and III are the indicators of mitochondrial function. The above results indicated that the structure and function of mitochondria in breast muscle were damaged.

Mitochondrial dysfunction could impair the redox state of mitochondria, the excessive ROS produced by H_2_O_2_ exposure caused cell damage through lipid peroxidation and protein oxidation [21]. The protein carbonyl and MDA are representative biomarkers of oxidative damage to muscle mitochondrial protein and lipid. The oxidation of lipid and protein have are considered the main causes of meat deterioration, which results in discoloration, low water holding capacity and loss of nutritional value in meat [4]. It was traditionally considered that the excessive ROS were eliminated by antioxidant enzymes such as CAT, GSH-Px, and SOD, but the excessive increase of ROS destroyed the redox balance and inhibited the activities of antioxidant enzymes [2,8]. According to our results, the contents of protein carbonyl and MDA were higher in H_2_O_2_-treated mitochondrial samples, and the activities of T-AOC, T-SOD, and GSH-Px were decreased after H_2_O_2_ exposure in mitochondria, which indicated that the antioxidant enzyme defensive system was damaged under oxidative stress.

The damage of mitochondrial structure and function, and the destruction of redox state may lead to the disorder of calcium metabolism in muscle, which could affect the meat quality [22]. Meanwhile, previous study has shown that the excessive production of ROS would lead to the breakdown of calcium homeostasis [9]. The deregulation of intracellular calcium could affect metabolic systems and even lead to metabolic diseases. Therefore, the deeper understanding of the interaction between calcium signaling pathway and ROS may help clarify the relationship between meat quality and oxidative stress. At the level of intracellular and extracellular calcium exchange, TRPC induce calcium influx, PMCA and NCX release calcium to the extracellular matrix. Under oxidative stress, excessive ROS would damage the cell membrane potential and cause calcium overflow into the cytoplasm [9]. Furthermore, excessive ROS could inhibit the activity of NCX and lead to the influx of calcium into the cytoplasm [23]. In sarcoplasmic reticulum (SR), the major Ca^2+^ release channel is the ryanodine receptor (RyR) and the recovery channel is SERCA1 [9]. The Ca^2+^ in mitochondria mainly comes from the release of the extracellular matrix and SR. The increase of cytoplasmic Ca^2+^ enters the mitochondria through MCU. The activity of the MCU is restricted due to MMP depletion. As massive amounts of Ca^2+^ enter the mitochondria, mitochondria in order to eliminate Ca^2+^ mainly through the mitochondrial NCX [24]. SERCA1 and MCU contain many cysteines, thus making them good targets for redox modification. ROS could directly regulate the activities of SERCA1 and MCU by oxidizing thiol groups. Carbonylation is the main and common oxidative modification of proteins, which affects the functional and metabolic stability of modified proteins. Protein carbonylation is an irreversible oxidative modification of a protein by generating reactive carbonyl functional groups such as aldehydes, ketones, or lactams after the side chains of amino acid residues are attacked by ROS [25]. Oxidative modification leads to the attenuation of SERCA1, which hinders the removal of Ca^2+^ from the cytoplasm and promotes the accumulation of Ca^2+^ in cells [26]. Oxidative stress leads to oxidative modification of cysteine 97 subunit of MCU, which inhibits the activity of MCU and the entry rate of Ca^2+^ [27]. In this study, after H_2_O_2_ exposure, the contents of Ca^2+^ in cytoplasm and mitochondria was increased, the protein expression levels of SERCA1 and MCU were decreased, and the protein expression level of PMCA was increased. Additionally, after H_2_O_2_ exposure, the carbonylation level of MCU was increased. These data suggested that cytoplasmic calcium overload was attributed to the excessive calcium influx of extracellular matrix into the cytoplasm and the dysfunction of the SR channel, which was consistent with the findings of Ermak and Davies [28]. H_2_O_2_ exposure broke Ca^2+^ metabolism by inhibiting the expression levels of SERCA1 and MCU, inducing Ca^2+^ overload in mitochondria, and further damaging mitochondria.

The mitochondrial damage, ROS overproduction and calcium overload are known factors inducing apoptosis, which could cause mitochondrial outer membrane permeabilization, promote the release of apoptotic factors into the cytoplasm, induce caspase activation, and initiate apoptosis [17]. Apoptosis could destroy the structure of muscle cells, cause the degradation of cytoskeleton of muscle cells, and affect muscle water holding capacity and muscle tenderness. In recent years, ferroptosis, a newly identified mode of cell death, has become a research hotspot in the field of life science. Mitochondrial dysfunction, lipid peroxidation and ROS overproduction are the characteristic of ferroptosis [11]. Therefore, it is speculated that oxidative stress is closely related to ferroptosis, and there are few reports on ferroptosis in broilers. GPX4 is a key enzyme in the mitochondrial antioxidant system, and it is also the key enzyme to inhibit ferroptosis in cells. The function of GSH in the antioxidant system is mainly regulated by SLC7A11, inhibition of SLC7A11 could weaken the function of GSH and promote ferroptosis [29]. Under normal physiological conditions, iron is transferred into cells as trivalent iron by TFR1 and stored in ferritin heavy chain 1 (FTH1) as divalent iron. Under oxidative stress, cells would increase iron intake or reduce iron storage, resulting in iron overload in the iron pool, the overloaded Fe^2+^ could oxidize lipid to produce ROS, thus damaging organelles and leading to ferroptosis [11]. In this study, after H_2_O_2_ exposure, the number of apoptotic cells was increased, and the iron was accumulated. H_2_O_2_ exposure decreased the activity of GSH and the ratio of GSH/GSSG, increased the activity of GSSG. Gonzalez et al [30]. reached a similar conclusion, which suggests that oxidative stress could impair the integrity of mitochondrial membrane structure and cause iron overload in mitochondria, the excessive iron in mitochondria may accelerate the formation of ROS by Fenton reaction. After H_2_O_2_ exposure, the mRNA and protein expression levels of TFR1 were increased and the mRNA and protein expression levels of GPX4 were decreased. Meanwhile, in the H_2_O_2_-treated group, the mRNA expression levels of IREB2 and SLC7A11 were down-regulated. These findings suggested that oxidative stress induced by H_2_O_2_ impaired the function of GSH caused iron overload and activated the ferroptosis signal pathway. In conclusion, our results revealed that oxidative stress induced by H_2_O_2_ damaged the mitochondrial antioxidant system in the breast muscle of broilers, excessive ROS impaired calcium metabolism, resulting in an overload of Ca^2+^ in the mitochondria. The disorder of calcium metabolism further led to the mitochondrial dysfunction, which induced ferroptosis and resulted in the deterioration of broiler meat quality.

## Figures and Tables

**Table 1:** Composition and nutrient levels of the basal diets

Items	1 to 21 days	22 to 42 days
Ingredients (%)		
Corn	57.61	62.27
Soybean meal	31.00	23.00
Corn gluten meal^1)^	3.29	6.00
Soybean oil	3.11	4.00
Limestone	1.20	1.20
Dicalcium phosphate	2.00	2.00
L-lysine	0.34	0.35
DL-methionine	0.15	0.08
Salt	0.30	0.30
Premix^2)^	1.00	1.00
Calculated nutrient levels		
ME (MJ/kg)	12.56	13.19
CP (%)	21.10	19.60
Ca (%)	1.00	0.95
Available phosphorus (%)	0.46	0.39
Lysine (%)	1.20	1.05
Methionine (%)	0.50	0.42
Methionine+cysteine (%)	0.85	0.76
Analyzed nutrient levels		
CP (%)	20.84	19.23
Ca (%)	1.03	0.99
Total phosphorus (%)	0.64	0.61

ME, metabolizable energy.

1) The crude protein (CP) content was 60%.

2) Premix provided per kilogram of diet: vitamin A, 12,000 IU; cholecalciferol for vitamin D_3_, 2,500 IU; DL-α-tocopheryl acetate for vitamin E, 20 IU; menadione sodium bisulfate, 1.3 mg; thiamin, 2.2 mg; riboflavin, 8.0 mg; nicotinamide, 40 mg; choline, 400 mg; calcium pantothenate, 10 mg; pyridoxine HCl, 4 mg; biotin, 0.04 mg; folic acid, 1 mg; vitamin B_12_ (cobalamin), 0.013 mg; Fe (from ferrous sulfate), 80 mg; Cu (from copper sulfate), 8.0 mg; Mn (from manganese sulfate), 110 mg; Zn (from zinc sulfate), 60 mg; I (from calcium iodate), 1.1 mg; Se (from sodium selenite), 0.3 mg.

**Table 2:** The primer sequences used for real-time polymerase chain reaction analysis

Gene	Genbank number	Primer sequence (5′→3′)	Product size (bp)
*TFR1*	NM_205256.2	Forward: GTTATCGTGGACGAATCGAGC	217
		Reverse: ACACCTACCCTCCACCTCAA	
*FTH1*	NM_205086.1	Forward: TACGCCTCCTACGTGTACCT	165
		Reverse: CTGCAAGAAGATGCGTCCAC	
*IREB2*	NM_001031454.1	Forward: TGCCCACGTCTTGCTGTATT	165
		Reverse: ATCACAGCATCGTTACCCCG	
*SLC7A11*	XM_426289.5	Forward: GACTGGTAGTTGCTGGCTTGA	295
		Reverse: TCCTCTGACGGGACAACTTC	
*GPX4*	AF498316.2	Forward: GGTGAGGCAGACCCGAAGA	134
		Reverse: CGTTTCCAGTGGGTTTATTTCA	
*GAPDH*	NM_204305.1	Forward: TCAAATGGGCAGATGCAGGT	291
		Reverse: TGATGGCATGGACAGTGGTC	

*TFR1*, transferrin receptor 1; *FTH1*, ferritin heavy chain 1; *IREB2*, iron responsive element binding 2; *SLC7A11*, solute carrier family 7 member 11; *GPX4*, glutathione peroxidase 4; GAPDH, glyceraldehyde-3-phosphate dehydrogenase.

**Table 3:** Effects of H_2_O_2_ exposure on the meat qualities of breast meat in broilers

Items	Treatments^1)^			SEM	p-value

	Control	Saline	H_2_O_2_		
pH_45 min_	6.54	6.57	6.53	0.04	0.944
pH_24 h_	5.88^a^	5.92^a^	5.82^b^	0.02	0.034
Redness (a*)	2.61	2.43	2.72	0.09	0.470
Yellowness (b*)	5.35	5.34	5.40	0.19	0.993
Lightness (L*)	42.59	41.95	41.08	0.35	0.228
Drip loss (%)	1.67^b^	1.71^b^	2.34^a^	0.12	0.028
Cooking loss (%)	8.58	7.30	8.21	0.31	0.237
Shear force (N)	28.34^b^	25.85^b^	34.21^a^	1.26	0.017

SEM, standard error of the mean; BW, body weight.

The results are represented as the mean value with pooled SEM (n = 6).

1) The control group was the non-injected treatment. The saline group: birds were injected with physiological saline buffer (0.75%) with a dosage of 1.0 mL/kg BW. The H_2_O_2_ group: birds were given an injection of 10.0% H_2_O_2_ with a dosage of 1.0 mL/kg BW.

a,b Means in a row without a common superscript letter significantly differ (p<0.05).

## References

[1] Petracci M, Cavani C (2012). Muscle growth and poultry meat quality issues. Nutrients.

[2] Chen ZD, Xing T, Li JL, Zhang L, Jiang Y, Gao F (2022). Oxidative stress induced by hydrogen peroxide promotes glycolysis by activating CaMKK/LKB1/AMPK pathway in broiler breast muscle. Poult Sci.

[3] Lin H, Decuypere E, Buyse J (2006). Acute heat stress induces oxidative stress in broiler chickens. Comp Biochem Physiol A Mol Integr Physiol.

[4] Falowo AB, Fayemi PO, Muchenje V (2014). Natural antioxidants against lipid–protein oxidative deterioration in meat and meat products: a review. Food Res Int.

[5] Chen XX, Zhang L, Li JL, Gao F, Zhou GH (2017). Hydrogen peroxide-induced change in meat quality of the breast muscle of broilers is mediated by ROS generation, apoptosis, and autophagy in the NF-kappa B signal pathway. J Agric Food Chem.

[6] Chen X, Gu R, Zhang L (2018). Induction of nuclear factor-kappa B signal-mediated apoptosis and autophagy by reactive oxygen species is associated with hydrogen peroxide-impaired growth performance of broilers. Animal.

[7] Rhoads RP, Baumgard LH, Suagee JK, Sanders SR (2013). Nutritional interventions to alleviate the negative consequences of heat stress. Adv Nutr.

[8] Chen ZD, Xing T, Li JL, Zhang L, Jiang Y, Gao F (2021). Hydrogen peroxide-induced oxidative stress impairs redox status and damages aerobic metabolism of breast muscle in broilers. Poult Sci.

[9] Gorlach A, Bertram K, Hudecova S, Krizanova O (2015). Calcium and ROS: a mutual interplay. Redox Biol.

[10] Dixon SJ, Lemberg KM, Lamprecht MR (2012). Ferroptosis: an iron-dependent form of nonapoptotic cell death. Cell.

[11] Imai H, Matsuoka M, Kumagai T, Sakamoto T, Koumura T, Nagata S, Nakano H (2016). Lipid peroxidation-dependent cell death regulated by GPx4 and ferroptosis. Apoptotic and non-apoptotic cell death. Current Topics in Microbiology and Immunology.

[12] Pan XN, Zhang L, Xing T, Li JL, Gao F (2020). The impaired redox status and activated nuclear factor-erythroid 2-related factor 2/antioxidant response element pathway in wooden breast myopathy in broiler chickens. Anim Biosci.

[13] Fellenberg MA, Speisky H (2006). Antioxidants: their effects on broiler oxidative stress and its meat oxidative stability. World Poult Sci J.

[14] Parrish FC, Selvig CJ, Culler RD, Zeece MG (1981). CAF activity, calcium concentration, andy the 30,000-dalton component of tough and tender bovine longissimus muscle. J Food Sci.

[15] Lu Z, He XF, Ma BB (2017). Chronic heat stress impairs the quality of breast-muscle meat in broilers by affecting redox status and energy-substance metabolism. J Agric Food Chem.

[16] Mujahid A, Yoshiki Y, Akiba Y, Toyomizu M (2005). Superoxide radical production in chicken skeletal muscle induced by acute heat stress. Poult Sci.

[17] Brentnall M, Rodriguez-Menocal L, De Guevara RL, Cepero E, Boise LH (2013). Caspase-9, caspase-3 and caspase-7 have distinct roles during intrinsic apoptosis. BMC Cell Biol.

[18] Cadenas E, Davies KJA (2000). Mitochondrial free radical generation, oxidative stress, and aging. Free Radic Biol Med.

[19] Noeman SA, Hamooda HE, Baalash AA (2011). Biochemical study of oxidative stress markers in the liver, kidney and heart of high fat diet induced obesity in rats. Diabetol Metab Syndr.

[20] Chance B, Sies H, Boveris A (1979). Hydroperoxide metabolism in mammalian organs. Physiol Rev.

[21] Reid MB (2008). Free radicals and muscle fatigue: Of ROS, canaries, and the IOC. Free Radic Biol Med.

[22] Soares AL, Ida EI, Miyamoto S (2003). Phospholipase A2 activity in poultry PSE, pale, soft, exudative, meat. J Food Biochem.

[23] Kaneko M, Matsumoto Y, Hayashi H, Kobayashi A, Yamazaki N (1994). Oxygen free radicals and calcium homeostasis in the heart. Mol Cell Biochem.

[24] Palty R, Hershfinkel M, Sekler I (2012). Molecular identity and functional properties of the mitochondrial Na+/Ca2+ exchanger. J Biol Chem.

[25] Zheng J, Bizzozero OA (2010). Traditional reactive carbonyl scavengers do not prevent the carbonylation of brain proteins induced by acute glutathione depletion. Free Radic Res.

[26] Strosova MK, Karlovska J, Zizkova P (2011). Modulation of sarcoplasmic/endoplasmic reticulum Ca2+-ATPase activity and oxidative modification during the development of adjuvant arthritis. Arch Biochem Biophys.

[27] Lee SK, Shanmughapriya S, Mok MCY (2016). Structural insights into mitochondrial calcium uniporter regulation by divalent cations. Cell Chem Biol.

[28] Ermak G, Davies KJA (2002). Calcium and oxidative stress: from cell signaling to cell death. Mol Immunol.

[29] Wang H, An P, Xie EJ (2017). Characterization of ferroptosis in murine models of hemochromatosis. Hepatology.

[30] Gonzalez PM, Piloni NE, Puntarulo S, Catala A (2012). Iron overload and lipid peroxidation in biological systems. Lipid peroxidation.

